# Valuing the Multiple Impacts of Household Food Waste

**DOI:** 10.3389/fnut.2019.00143

**Published:** 2019-09-04

**Authors:** Michael von Massow, Kate Parizeau, Monica Gallant, Mark Wickson, Jess Haines, David W. L. Ma, Angela Wallace, Nicholas Carroll, Alison M. Duncan

**Affiliations:** ^1^Department of Food, Agricultural and Resource Economics, University of Guelph, Guelph, ON, Canada; ^2^Department of Geography, Environment, and Geomatics, University of Guelph, Guelph, ON, Canada; ^3^Department of Family Relations and Applied Nutrition, University of Guelph, Guelph, ON, Canada; ^4^Department of Human Health and Nutritional Sciences, University of Guelph, Guelph, ON, Canada

**Keywords:** food waste, household waste, composition audit, nutrition loss, environmental impact, economic cost

## Abstract

The Commission for Environmental Cooperation (CEC) has estimated that Canadian households waste 85 kg of food per person annually. Food waste has become an increasingly common focus for policy, regulation, interventions, and awareness-raising efforts in Canada. However, there is still a relative dearth of data to inform such decision-making processes or to provide narratives to contextualize behavior change efforts. In this paper, we describe the results of an uncommonly detailed observational study of household food waste. A total of 94 families with young children living in Guelph, Ontario chose to participate in this study. Over the course of multiple weeks, we collected data on their food purchases, food consumption, and waste generation. All three streams of waste (garbage, recycling, and organic waste) were audited and the food type, degree of avoidability, and weight of each individual component of the organic waste stream was recorded. Using this highly granular data set, we found that the average household in our study generated approximately 2.98 kg of avoidable food waste per week. This estimate was then contextualized in terms of economic losses (dollar value), nutritional losses (calories, vitamins, and minerals) and environmental impacts (global warming potential, land, and water usage). In short, weekly avoidable food waste per household was calculated to be equivalent to $18.01, 3,366 calories, and 23.3 kg of CO_2_. These multiple valuation frameworks, which are based in detailed observations of family food behaviors rather than estimations derived from system-wide data, will enable more informed and urgent conversations about policy, programming, and interventions in order to reduce the volume of wasted food at the consumer level.

## Introduction

At the international scale, there has been a relatively recent increase in attention to food waste in both research and policy (1–4), suggesting that conversations about this topic have gained prominence and momentum in our collective consciousness. Reducing food waste was included as one of the United Nations Sustainable Development Goals under the priority of “Responsible Consumption and Production” (5). The Food and Agriculture Organization has issued reports and produced public commentary on food loss and waste over the past decade (6), and the European Union has prioritized food waste measurements and interventions since 2012 under the FUSIONS and REFRESH projects (7, 8). In Canada, food waste has recently become the subject of municipal, provincial, and national policy discussions. The creation of multi-stakeholder organizations to inform food waste policy-making and intervention design [e.g., see (9–11)] is another indicator of rising attention to food waste as an issue of concern in the Canadian context.

In order to meaningfully address this critical issue which has important environmental, economic, and nutritional consequences, food waste policy-making and intervention-testing must be supported by high-quality evidence. In this study, we discuss the results of a highly granular waste composition audit conducted with 94 households in Guelph, Ontario. We highlight the uniqueness of our data set and discuss the potential for using high quality data to inform the emergence of food waste discourses. We then analyze our data from different vantage points in order to frame our results in terms of the economic losses, nutritional losses, and environmental impacts of household food waste in Canada. This analysis can be used to inform messaging choices for policy-making, advocacy activities, and educational and behavior-change interventions.

## Measurement and Characterization of Food Waste

Measuring and monitoring food waste generation is a challenging task. The Commission for Environmental Cooperation recently worked to create a comprehensive estimate for organic waste in Canada. Their methodology relied on the extrapolation of a limited number of composition audits from the residential and ICI (industrial, commercial, and institutional) sectors to generate national level data. Their final estimates suggest that Canada generates 12.6 million tons of organic waste that is sent to final disposal, and an additional 5.8 million tons of organics that are diverted to alternative treatment. They estimate that individuals generate approximately 85 kg of food waste per year in the residential sector (12). Using a different approach, Gooch et al. (13) used surveys, interviews, and secondary data to inform their estimates of food loss and waste (FLW) at different points in the Canadian food value chain. They estimate that 35.5 million metric tons of food are lost or wasted in Canada, including 11.2 million metric tons of avoidable food waste. They characterize this waste as representing 58.1% of the commodities entering the food system, at a cost of $49.5 billion. In this study, household food waste was not directly observed, but was estimated using aggregate food availability data from Statistics Canada.

Canadian municipalities often conduct audits of their residential waste streams in order to learn about their composition, which may include organic waste generation rates. However, these audits often collect aggregate data (rather than observations at the individual household level), and their methodologies can vary widely [see (10) for a food waste audit guide that would allow for improved cross-site data comparability]. There is little academic literature that systematically observes household food waste generation in Canadian contexts [for exceptions see (14, 15)]. Similarly, there are relatively few direct observations of organic waste in other parts of the world [see (16) for a detailed exception based in the UK]. Xue et al. (2) note that over half of the published articles in their systematic review of food waste across the supply chain relied on secondary data, whereas only about 20% of these studies used direct observations. Furthermore, the category of direct observations included self-report methods such as surveys and diaries: research shows that such observational methods can be unreliable and tend to underestimate food waste generation (17–19). We are unaware of any other research in Canada that systematically records each item of food waste generated at the individual household level, and we believe that our highly granular dataset enables a different framing of food waste as a policy-relevant issue.

## Building Story-lines: A Project of Environmental Discourse

How has food waste come to gain prominence and visibility in our modern world? In his classic analysis of environmental discourse, Hajer (20) observes that certain issues become emblematic at distinctive times, garnering public and political attention. Even when issues become emblematic, they are not necessarily coherent. For example, Hajer discusses how the issue of acid rain emerged as a topic pertaining to multi-disciplinary ecological understandings, economic implications, and the social and financial impacts of interventions and abatement techniques, as well as ethical discussions regarding blame and responsibility. Therefore, environmental discourses do not refer solely to the discussion of environmental science, but also to related issues of interest to diverse actors in society.

Hajer's analysis focuses on the enabling capacity of discourses, and the ability of individuals to strategically deploy discursive strategies. In other words, discursive formations can be influenced and designed. He argues that the struggle for discursive hegemony is a political project whereby different actors “try to secure support for their definition of reality” (p.59). One such discursive approach is the creation of story-lines:

A story-line, as I interpret it, is a generative sort of narrative that allows actors to draw upon various discursive categories to give meaning to specific physical or social phenomena. The key function of story-lines is that they suggest unity in the bewildering variety of separate discursive component parts of a problem like acid rain…Finding the appropriate story-line becomes an important form of agency [(20), p.56].

An indicator that a discourse has become hegemonic is that it has become institutionalized; that is, the discourse is manifest in institutional arrangements, such as policies, institutional structures, or formal practices. The dominant discursive framings of environmental issues thus have implications for how these issues are perceived by the public, which institutional actions are deemed appropriate as interventions, and whether such issues are seen as actionable in the first place (21).

With respect to food waste in Canada, we have seen the proliferation of discursive framings of these issues in the past 5 years. We believe that this is an important moment for designing public messages that are evidence-based, action-oriented, and relevant to public policymakers. In this article, we draw upon our highly granular food waste audit data to suggest different means of communicating the impacts of food waste in health, economic, and environmental terms to both policymakers and the public at large. In essence, we are suggesting story-lines that “illustrate where [our] work fits into the jigsaw” [(20), p.63]. In the following section, we map out some of the other puzzle pieces that constitute the sometimes incoherent realm of food waste discourses in the contemporary Canadian context [also see (22) for a discourse analysis of food waste in the United Kingdom].

## Existing Food Waste Story-lines in Canada

The federal government in Canada has indicated its interest in addressing food waste, although it has not yet issued policies or regulations that would institutionalize this commitment. Environment and Climate Change Canada (the federal environment ministry) has recently taken leadership on the food waste file, convening multi-stakeholder workshops and commissioning reports on this topic. While they are in the process of articulating their position on this topic, they have not been the primary source of environmental discourse on food waste to date. Similarly, in a study on the prospective creation of a Canadian food policy, the House of Commons Standing Committee on Agriculture and Agri-Food made recommendations that “the Government, in conjunction with all members of the supply chain, establish education tools and take action to reduce industry food loss and consumer food waste,” and also that the Government work with community groups and NGOs to address a suite of food-related issues, including food loss and waste (23). The details of these mechanisms were not clearly articulated in the study, and the food policy-making process is still underway at the time of writing.

Provincial governments in Canada have primary responsibility for waste management legislation in Canada, and some provinces have foregrounded food waste in their policy and regulations. For example, Prince Edward Island and Nova Scotia have banned organic wastes from landfills, and Quebec is implementing a staged ban as well (12). Although they have been responsible for legislation that enables the diversion of organic wastes, provincial governments have otherwise not been as active in the generation of discourse around the impacts of food waste or interventions to address this issue. An exception is the province of British Columbia, which has developed a suite of toolkits and resources to enable different actors to prevent and reduce their food waste (24). Ontario's Ministry of the Environment, Conservation, and Parks (MECP) recently released a Discussion Paper entitled “Reducing Litter and Waste in Our Communities” that addresses food waste, among other topics. Under the heading “Build a culture of food waste avoidance,” the document suggests the following:

…the province will work with partners to develop educational tools and resources, including guidance on the implementation of the policy statement, to support more standardized promotion and education outreach (e.g., best practices for meal planning and food storage, including tips on how to extend the life of food, such as freezing food where appropriate and safe) [(25), p.16]

This framing of food waste focuses on influencing individual-level behaviors as a policy mechanism. The Discussion Paper iterates an interest in developing a landfill ban for organic materials as a more systemic intervention, and also broaches the expansion of organics diversion programs “where it makes sense” [(25), p.18]. This document alludes to the potential for food rescue as a solution for food waste, which is discussed as a discursive phenomenon below. Overall, the current Ontario government's perspective on food waste as an environmental issue is that win-win solutions are possible: “Avoiding food waste, rescuing surplus food, and diverting unavoidable food and organic waste is both good for the environment and good for business” [(25), p.15].

Much of the policy and planning work on food waste in Canada occurs at the scale of municipal or regional governments. Some municipalities have their own local communications and awareness-raising programs, such as York Region's Good Food Program. This campaign focuses on healthy eating and food skills messaging to encourage residents to eat the food that they have already purchased, and thereby reduce food waste (26). Metro Vancouver's “Hey! Food Isn't Garbage!” campaign was designed to encourage diversion of food scraps and was rolled out in conjunction with a regional Organics Disposal Ban and increased residential access to source separated organics collection programs (27). Municipalities have also worked together to create story-lines about waste. For example, the Ontario Food Collaborative is a group of municipal waste managers and public health staff working to address food waste and shift local cultures around this issue. They recently published a Food Waste Audit Guide (discussed above) meant to encourage municipalities to measure and monitor food waste generation in order to better prevent it. They also published a Food Waste Reduction and Healthy Eating Communications Strategy whose goal is “To inform, motivate, and empower people to live a more sustainable lifestyle by providing education, tools and resources to promote and support healthy eating and food waste prevention and reduction” [(11), p.6].

The National Zero Waste Council (NZWC) is a multi-stakeholder initiative that was initiated by the regional government of Metro Vancouver with the objective of advancing a waste prevention agenda across Canada. This organization includes major Canadian municipalities, businesses, and non-profits. Notably, the NZWC has licensed Love Food Hate Waste—a successful awareness-raising campaign designed in the United Kingdom by the Waste and Resources Action Programme (WRAP)—for use in Canada. Municipalities, provinces, and businesses can sign on as partners to access the campaign materials and social media platforms, which include messages around the reduction of household food waste. The campaign focuses on food skills (i.e., the provision of recipes, tips, and the tagline “Plan it Out, Use it Up, Keep it Fresh”), and also conveys messages about the economic costs of household food waste (“Wasted food costs an average Canadian household over $1100 per year”), the scale of household food waste (“Over 60% of household food waste in Canada is avoidable”), and information about the carbon footprint of this waste [“Reducing 1 ton household food waste = 1 car off the road each year”; (28)].

The NZWC also published “A Food Loss and Waste Strategy for Canada” in 2017. This document frames the problem of food waste as follows:

The strategy calls for the federal government to publicly announce a national target and takes a systems approach that aims to change practices and policies at key leverage points along the value chain and in the mandates of governments, as well as encourage new behaviors. It is anchored by three broad objectives: Prevent food waste from occurring in the first place; Recover safe and nutritious food for people and food scraps for animals; and Recycle energy and nutrients from the remaining, unavoidable food waste [(9), p.7]

This strategy thus frames food waste as a systemic and policy-relevant issue that requires attention across the food value chain. It references the environment and economic costs of food waste and alludes to the potential for food recovery from this waste stream.

Another major source of food waste discourse in Canada is a series of reports generated by Value Chain Management International (13, 29, 30), the most recent of which was commissioned by the non-profit food rescue organization Second Harvest. These often-referenced reports investigate the source and scale of food waste in Canada, and the messaging has changed over time as new data have come to light. The 2010 report focused on the negative economic and environmental repercussions of an estimated $27 billion in food loss and waste in Canada, noting that “While the majority of food waste occurs at the consumer level, improving the management of agri-food value chains would have the greatest long-term impact on reducing food waste” (30). In 2014, the estimate of the value of food loss and waste was adjusted to $31 billion, it was noted that consumers were still the leading source of food waste, and a main theme in food waste generation was adversarial relationships along the food value chain (30). The 2019 report used different methods to generate their estimates for food loss and waste, which was reported as the equivalent of $49.5 billion. Notably, the increase in food waste was observed at the manufacturing and processing stages, which then displaced consumers as the leading source of waste. This most recent report identifies broad structural and cultural causes for the high volume of food waste observed in Canada:

The root causes of the FLW that occurs in Canada include a culture of accepting waste. A direct correlation can be drawn between some business and governmental decisions and the creation of avoidable FLW. Other root causes of FLW include the true cost of FLW not being internalized by industry and consumers. In addition, there is no common template for redistributing food that would otherwise go to landfill or non-food use [(13), p.6].

In this discursive framing, food waste is posited as a series of structural and cultural short-comings that span the food system. It is a lost opportunity that negatively impacts multiple stakeholders.

In the Canadian context, food security has often been invoked when discursively defining food waste as a problem. For example, the NZWC called for a tax credit for corporate food donations to incentivize the diversion of wasted food to non-profit organizations in 2015–6; this strategy was framed as one among many needed to prevent the generation of food waste. A business case study prepared by the Conference Board of Canada for the NZWC listed “Increasing, Improving, or Enhancing Household Food Security” as one of the benefits of a donation tax incentive, alongside environmental and economic benefits [(31), p. 15]. Many high-profile food security advocates in Canada responded to the framing of food waste as a food security issue, pointing out that because food security is primarily an issue of inadequate income, food donation can never address the root causes of hunger in Canada. They also noted that incentivizing large-scale donations of wasted food discourages systemic interventions that prevent or reduce this waste stream, and that this approach may overwhelm under-resourced non-profit organizations with high volumes of varying quality food (32–35). Proponents of the tax credit (including the NZWC) subsequently deemphasized the food security argument in acknowledgment of the issues raised by food security advocates, noting that the prevention of food waste was always their primary aim. For example, in September 2016, the Federation of Canadian Municipalities (FCM) expressed their support for the tax credit, but did not mention food security as a motivator for this endorsement: “That FCM support the National Zero Waste Council's food waste reduction federal tax incentive proposal … thereby helping reduce food waste, lower municipal costs for waste disposal and decrease the environmental impact of food waste” (36).

While food waste discourse in Canada is not coherent, there are some common themes that emerge in the messaging from key influencers. Food waste is described as a multifactorial problem: its impacts are environmental, economic, social, and health related. However, health issues are usually framed around the benefits of eating commonly wasted food, rather than focusing on the nutrients that are lost when food is wasted. Food waste occurs at multiple sites, and so many actors can be read as responsible for its generation. Some framings focus on preventing food waste, while others see this as a problem to be mediated via reduction or treated through diversion and composting. The tenor of discussion surrounding interventions varies depending on which parties are responsibilized for food waste. For example, interpretations that focus on the consumer as the appropriate site of intervention tend to focus on skill building and education, whereas structural analyses focus on policy mechanisms and regulatory interventions (these messages are often not mutually exclusive within a given discourse, however). Different discourses often reference the potential for synergies, such as the ability to save money and reduce environmental impact at the same time, or to improve the quality of one's diet while also reducing pressure on municipal waste management infrastructure.

Welch et al. (22) conducted a similar discourse analysis of food waste in the United Kingdom, finding that the discourse coalition that has emerged there has assumed the dominant framing of food waste is one of “responsibility distributed throughout the production–consumption system” (p.1). They also observed narratives framing food waste as a “perfect storm” of issues, including environmental, social, and economic impacts. These authors argue that the discourse collation that has emerged in the United Kingdom around food waste has reached the stage of discursive hegemony. In contrast, food waste discourse is still emergent in Canada, and there is room to shape the story-lines that are framing the public conversation about this issue. In the following sections, we mobilize our household food waste audit data with the aim of informing some of these discursive framings. Our goal is to provide framings that may help to convince diverse policy-makers of the gravity of this issue, that may educate and motivate consumers to change their individual behaviors and to advocate for civic action on food waste, and that may inform future research on the effectiveness of different messages, as well as discursive analyses of “wicked” environmental problems in the current age. The following analysis focuses on providing evidence for the key discursive framings of food waste that are already at play in Canada, including the economic, health / nutritional, and environmental impacts of avoidable household food waste.

## Materials and Methods

This study took place in the city of Guelph, Ontario, and was conducted in accordance with the University of Guelph's Research Ethics Board protocols. This study was carried out as part of the Family Food Skills Study, a cross-sectional study that aimed to examine family food behaviors and assess their impact on family diets. The study reported in this paper added a food waste composition audit to the Family Food Skills protocol to assess the relationship between family food skills, food behaviors, and food waste. Data reported in this paper includes results from the weekly household waste audits and results from the analysis of receipts collected for all food purchases, including grocery stores and meals purchased outside of the home.

Eligibility for this study required that families have at least one child between 2 to 8 years of age and that parents have no prior nutrition or food training. Recruitment took place at daycares and community health centers as well as through social media and word of mouth. As an incentive for voluntary participation, each family was offered a $100 grocery gift card. Prior to beginning the study, the study team performed home visits to each family in order to answer questions and explain expectations. Data was collected from 54 families in 2017 and 40 families 2018, for a total of 94 participating households.

The household waste audits were conducted over four consecutive weeks in 2017 and three consecutive weeks in 2018 over the summer. The shorter audit period in 2018 was driven by logistical constraints (i.e., availability of auditors and a holiday long-weekend, which can change waste behaviors). Waste was collected each week on the day the family would have expected their regular waste pick-up by the municipality. All three waste streams were collected, including source-separated organics, recycling, and residual garbage. Families who had a home composter were asked to place all of their organics in the green bin for the weeks that they were being audited. In this case, we were unable to observe food waste that was disposed of by other means (i.e., sink garburators, fed to animals etc.). It is also possible that the waste audits failed to capture some liquid items from the dairy group (i.e., milk, yogurt) as these products tend to be disposed of in the sink rather than in garbage or organics bins. Collected waste was then taken to a central municipal facility where it was audited to evaluate food waste volume and composition.

Auditors identified and weighed each individual food item found in any of the three streams. Waste was categorized into six broad categories based on the criteria used by the Guelph Food Waste Research Group in previous work in Guelph. These categories were adapted from WRAP Household Food Waste Collections Guide^1^. The six categories are: fruits and vegetables, meat and fish, grains and cereals, dairy (milk, cheese, and eggs), fats and sugars, and other (primarily coffee grounds and tea). The categories are also divided into avoidable (could have been eaten at some point), unavoidable (inedible portions of foods), and possibly avoidable (could be eaten but some people choose not to, e.g., potato peels). Our focus for this paper is on food that could have been eaten, and so we aggregated the avoidable and possibly avoidable categories.

Food scraps that were already mid-decomposition or blended in with other food scraps were labeled as “Unidentifiable” or “Unknown [food group category].” We proportionally distributed the weights of the these unknown/unidentifiable foods into known food categories. For example, if a household had “asparagus” food scraps which constituted 10% of that household's known vegetable weight for that week, the “asparagus” category would receive 10% of that household's “Unknown Vegetable” category. For composite meals involving several different food items mixed together, we first attempted to sort out the individual foods from the component foods. When this proved to be impossible, the food item was labeled according to all components present. For example, a mixture of rice and broccoli that was thoroughly blended was categorized as “Rice and Broccoli.” These composite meals would then be listed in the food group of its primary component, in this case “Rice.”

Once each food item was categorized, we generated a list of 316 avoidable food items found in the waste streams. We compared the total mean weights of the 2017 and 2018 sub-samples using a Mann-Whitney test and found that these were comparable sub-groups that could be combined for subsequent analysis.

## Quantification Methodology

We subsequently characterized the economic, nutritional, and environmental footprint of the avoidable food waste observed in the audits. For the economic analysis, the dollar value for each avoidable food item by weight was calculated using receipt data collected during the study. Any food items for which receipt data was not available (~12% of the data) were searched on the website of the grocery chain with the largest market share in Ontario. This store also represented a significant portion of the receipt tapes submitted. In some cases, weights were not available for the products and instead were purchased by units. When this occurred, the online weight converter at Hannaone.com^2^ was used to estimate the weight of unit-based foods.

The Canadian Nutrient File (CNF), created by Health Canada, is the standard reference food composition database outlining the amount of nutrients in foods commonly consumed in Canada. It is a comprehensive, computerized, bilingual database with information on up to 152 nutrients in over 5,690 foods^3^. The CNF was used to calculate wasted nutrient values in the avoidable waste observed in this study. During the audit process, many food items were labeled somewhat generically in order to facilitate sorting and characterization of food items (e.g., “bread”). When the word “bread” was entered in the online search criteria, 145 entries were retrieved and ranged in both form and nutritional value. In cases like this where the exact food item was unclear, we selected an entry that represented a mid-point with regard to nutritional quality. Versions of the composite meals observed in the audit were typically available in the CNF.

The selection of nutrients for the nutritional analysis was based on two criteria: nutrients that carry a daily value recommendation in Canada, and nutrients that are below recommended intake levels among Canadians. According to Health Canada, the prevalence of inadequate intakes among adults is highest for vitamin A, vitamin D, magnesium and calcium. Of additional concern are vitamin B12, fiber and vitamin C (37). Among Canadian children, fiber, calcium and vitamin D are also listed as nutrients of concern (38). Energy (kcal) was also included in this analysis. Health Canada suggests that on average, adults and youth (age 13 and older) require approximately 2,000 kcal per day and children (ages 4–12) need 1,500 kcal per day (39).

Daily values for the selected nutrients are publicly available and can be observed in the nutrition facts table required by law on most packaged foods in Canada. Current daily values in Canada are based on the recommended daily intake for vitamins and minerals as well as reference standards for various nutrients. Daily value suggestions have been calculated for infants (aged 6 months to 1 year), children (aged 1 to 4 years) and adults (any other case) (40).

Once the unique code numbers for all 316 food products were recorded, data from the CNF was used to generate nutrient profiles for every food item. The total lost nutritional value for each of the study households was calculated by multiplying the total weight of each item wasted by the amount of each nutrient listed for that food [i.e., Nutrient Loss = Food Waste Amount (grams) × Nutrition Concentration (nutrient amount/gram of edible food)].

There are several different assessments that could have been used to determine the environmental impacts of avoidable food waste. Based on the availability of data and an evaluation of measures used in common environmental discourse, three areas of environmental impact were prioritized. These areas were carbon dioxide (CO_2_) produced (i.e., global warming potential), land usage, and water usage. Global warming potential (GWP) is frequently used in the life-cycle-assessment (LCA) literature as a means of measuring the relative environmental impact of food production and waste. This is done by estimating how much CO_2_ is produced to not only grow, but also distribute agri-food products along their value-chain. Because LCA studies rely on very particular data for specific agri-food products, they are typically only concerned with a few commodities or classes of commodities. These estimates tend to be very region-specific. Furthermore, LCA studies face a “boundary problem” whereby the value-chain beginning and end for commodities is unclear. For example, there is inconsistency in determining the end of the environmental impact of a processed agri-food commodity depending on whether the LCA finishes once the product reaches the processor, retailer, or end-user.

Meta-analysis papers in the LCA literature are useful in this regard because they can combine and assess these varied studies to generate average estimates of environmental impacts for different food products. An LCA meta-analysis study conducted by Clune et al. (41) contains GWP estimations for over 150 food categories. This paper outlines estimations of the kg of CO_2_ produced per kg of edible food product. The authors convert the various boundaries of the LCA studies to a common benchmark of “farm to regional distribution center” (41). Two tables from this study are especially useful. Table 4 contains GWP statistics for animal products, proteins, and aggregate produce categories, while Table 5 contains GWP statistics for many specific food items. The total 316 avoidable food items in our audit data were matched with the most appropriate food item or food category in Tables 4 and 5 from Clune et al. (41). This matching provided each avoidable food waste item with a GWP figure and thus an estimate of CO_2_ produced per kg of food. While the tables in Clune et al. (41) include both mean and median GWP values, we only imported the median values to limit the impact of outliers.

Along with GWP, land usage is also commonly evaluated in LCA studies. While CO_2_ dispersion is esoteric and may be difficult for individuals to visualize, the amount of land used to produce food may be more concrete and relatable. Land usage is related to agricultural production yield, and the Food and Agricultural Organization (FAO) of the United Nations has data resources on yield figures for a wide variety of commodities around the world (6). Using data on crop yield from 1998 to 2018 in the FAOSTAT database, we generated the land in m^2^ necessary to produce a kg of over 150 food products. In the same manner as the GWP figures, the 316 avoidable food waste items were matched with the most appropriate food product from the FAOSTAT data which linked a land usage value to the unique food waste items in this study. The FAOSTAT yield data is mainly restricted to crops while data relating to animal products are mostly expressed in terms of total production per capita within a region. Thus, the remaining food waste items without land usage values were almost exclusively animal products. To fill in these gaps, we turned to another LCA meta-analysis study. Using the midrange values from Table 3 in Nijdam et al. (42), we estimated the land usage of the remaining protein-rich food items found in our audits.

Water usage is another common way of evaluating the environmental impact of different agri-food practices. The Water Footprint Network is an international group of academics and professionals which measures and tracks water usage statistics for many agri-food products and practices. They have created water usage databases for crops (43) and animal products (44) that are accessible for use and interpretation. Using these two databases, the m^3^ of water necessary to produce one kilogram of various food products was calculated. The avoidable food waste items from the audit data were linked with the water usage databases.

## Results

The average amount of total food wasted per household was 4.41 kg per week. This represents an average annual generation of 229.32 kg of total food waste per household. However, our focus in this analysis was on the avoidable and possibly avoidable quantities as this is the edible portion of the organic stream. The average avoidable food waste across the sample was 2.98 kg per week, which is consistent with our previous work in Guelph. The sample mean weights for the total basket and each category are shown in Table 1. The mean weights for the first and fourth quartiles of households are also reported. Fruits and vegetables made up approximately two thirds of the avoidable weight. Breads and cereals also made up a large portion (24%) of the avoidable weight.

**Table 1:** Composition of avoidable food waste.

	**Total items**	**Mean weight (grams)**	**Percent of total**	**1st quartile weight (grams)**	**4th quartile weight (grams)**
Full basket	316	2,978	100%	1,027	5,493
Fruits and vegetables	133	1,951	65.5%	693	3,671
Meat and fish	43	178	6.0%	66	322
Bread and cereals	74	722	24.2%	200	1,306
Dairy and eggs	17	63	2.1%	33	89
Fats and sugars	18	16	0.5%	4	23
Other	31	48	1.6%	31	83

**Figure 1:**
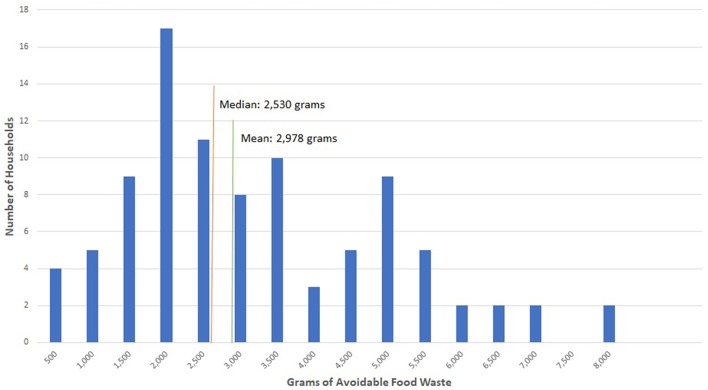
Household frequency of weekly avoidable food waste weight.

There was considerable variability between the waste generation rates of households. Figure 1 shows a histogram of weekly avoidable household food waste. The mean for the entire sample was 2.98 kg but the median was 2.53 kg. There were a small number of high waste households that increased the sample averages. There was also considerable variation within households from week to week.

**Figure 2:**
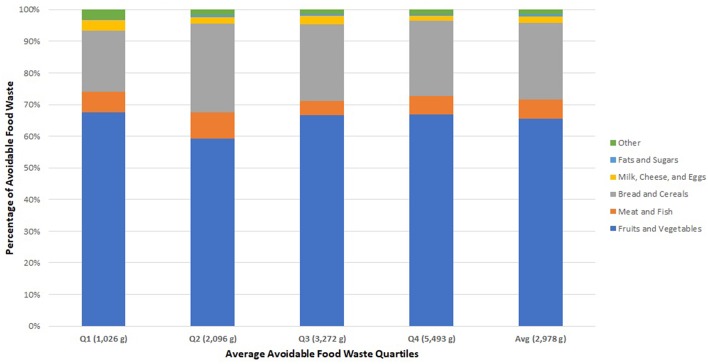
Comparison of avoidable food waste composition between quartiles.

The distribution of waste by categories across the four quartiles and on average is shown in Figure 2. While there was considerable variation from house to house and significant differences in total waste generation, the average composition of the organic waste stream did not change much as volume grew. Some households clearly disposed of more edible food, but the increase was consistent across all the food categories rather than being driven by a specific category.

**Figure 3:**
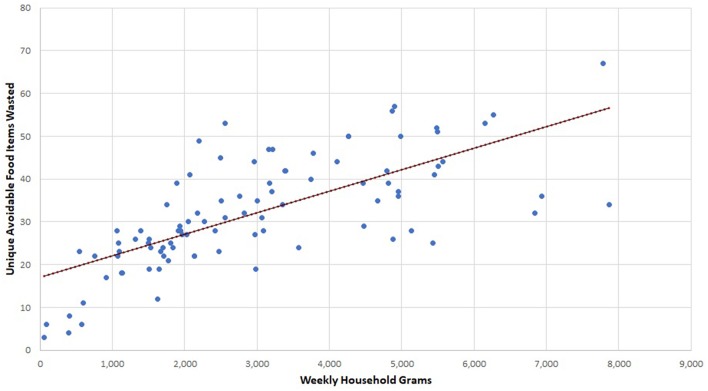
Avoidable food waste variety and avoidable food waste weight.

There was a relationship between the variety of food items discarded and the total weight of avoidable food waste (Figure 3). Our analysis yielded a Spearman correlation coefficient of 0.76 (*p* < 0.01) indicating that variety of food items discarded and food waste generation increase together, but there remains some unexplained variation. This concept merits future research.

The 25 items that represented the largest share of the avoidable food waste weight are presented in Table 2. These products represented almost 60% of the total weight of avoidable food waste generated by the sample. While there was a total of 316 edible food items in the total sample, after the top 25, no single item represented more than 1% of the total weight of the whole sample. However, it is critical to include the entire sample when calculating costs, lost nutrition and environmental impact, as the full-basket analysis generated substantially different results than an analysis of the top 25 items alone.

**Table 2:** Top 25 avoidable food waste items.

**Food item**	**Mean weight (grams)**	**Percentage of avoidable waste**	**Cumulative percentage**
Bread	268	9.0%	9.0%
Tomato	175	5.9%	14.9%
Apple	113	3.8%	18.7%
Watermelon	101	3.4%	22.1%
Potato	93	3.1%	25.2%
Pasta	78	2.6%	27.8%
Peach	72	2.4%	30.2%
Rice	70	2.4%	32.5%
Lettuce	66	2.2%	34.8%
Lemon	63	2.1%	36.9%
Pepper (incl. pepper top)	65	2.2%	39.1%
Chicken	60	2.0%	41.1%
Grapes	59	2.0%	43.1%
Cucumber	52	1.7%	44.8%
Broccoli stalks	51	1.7%	46.5%
Potato peels	46	1.6%	48.1%
Onion	45	1.5%	49.6%
Cabbage	44	1.5%	51.1%
Carrot	43	1.4%	52.5%
Banana	41	1.4%	53.9%
Celery	34	1.1%	55.0%
Broccoli	33	1.1%	56.1%
Tomato sauce	32	1.1%	57.2%
Carrot peels	31	1.0%	58.2%
Pear	31	1.0%	59.3%

## Economic Analysis

The total average household value of avoidable food waste was $18.01 per week (Table 3), or $936.52 per year. The median cost of avoidable food waste per household was $16.60 per week. The first quartile of households had an average cost of $6.47 per week while the fourth quartile had an average cost of $31.35 per week.

**Table 3:** Economic value of avoidable food waste.

	**Mean weight (grams)**	**Cost (CAD)**	**Percent of total**	**1st quartile dollars (CAD)**	**4th quartile dollars (CAD)**
Full basket	2,978	$18.01	100.0%	$6.47	$31.35
Fruits and vegetables	1,951	$9.36	52.0%	$3.34	$17.06
Meat and fish	178	$2.29	12.7%	$0.84	$4.37
Bread and cereals	722	$4.52	25.1%	$1.30	$7.39
Dairy and eggs	63	$0.71	3.9%	$0.43	$0.88
Fats and sugars	16	$0.12	0.7%	$0.08	$0.15
Other	48	$1.01	5.6%	$0.49	$1.51

Fruits and vegetables represented a lower proportion of total cost than they did of total weight, although they still represented more than 50% of the total cost of avoidable food waste. Meat and fish represented a small proportion of the total weight (6%) but a much larger proportion of total cost (13%). One would expect that more expensive items would be managed with more care and be wasted less, which appears to be the case in our sample.

**Figure 4:**
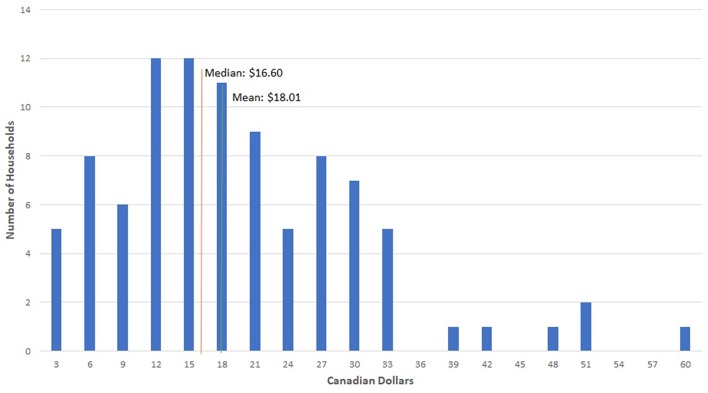
Household frequency of avoidable food waste cost.

There was a strong linear relationship between volume of waste and total value of waste (Spearman correlation coefficient of 0.92; *p* < 0.01). Eighty-four percent of the variation in value was explained by total weight of wasted food, and total cost increased by approximately $5.70 per kilogram of avoidable food waste. There were a small number of high volume and value households that drove up the average weekly cost of wasted food (Figure 4).

## Nutritional Analysis

**Figure 5:**
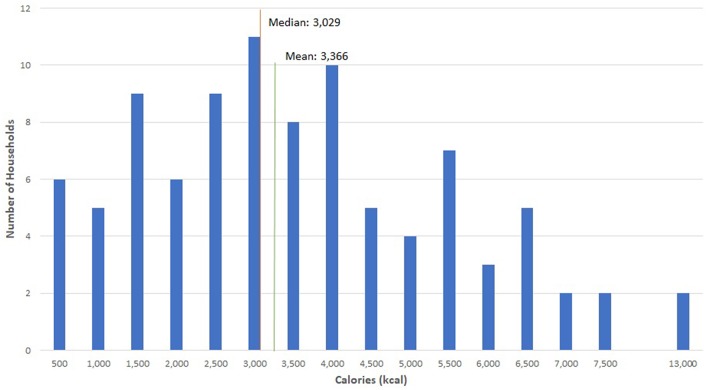
Household frequency of avoidable food waste calories.

The average household wasted 3,366 kcal per week (Table 4) or the equivalent of 175,032 kcal annually. This quantity of weekly food waste weight represents the suggested recommended daily caloric intake for 1.7 adults or 2.2 children. In other words, the average household could have provisioned an additional five adult meals or almost seven child meals per week based on the edible items they wasted. Annual avoidable food waste represents the suggested daily caloric intake for an adult for 88 days of the year, and for a child for 117 days. Calories appear to be relatively normally distributed with a slightly long tail to the right (Figure 5). We see a strong relationship between weight of avoidable food waste and calories. In this case, we observed a Spearman correlation coefficient of 0.87 (*p* < 0.01). The calories became more variable as weight increased and this likely reflects specific products that are consumed in these high waste households.

**Table 4:** Dietary composition of avoidable food waste.

	**Total**	**Daily servings adult**	**Daily servings child**	**1st quartile total**	**4th quartile total**
Energy (kcal)	3,366	1.7	2.2	1,191	5,993
Fiber (g)	64	2.3	4.6	21	115
Vitamin D (mcg)	50	2.5	3.3	12	113
Vitamin B12 (mcg)	2	1.0	2.8	1	5
Vitamin C (mg)	434	4.8	28.9	155	749
Vitamin A (mcg)	1,729	1.9	5.8	596	3,312
Calcium (mg)	1,192	0.9	1.7	403	2,061
Magnesium (mg)	675	1.6	8.4	218	1,190

**Figure 6:**
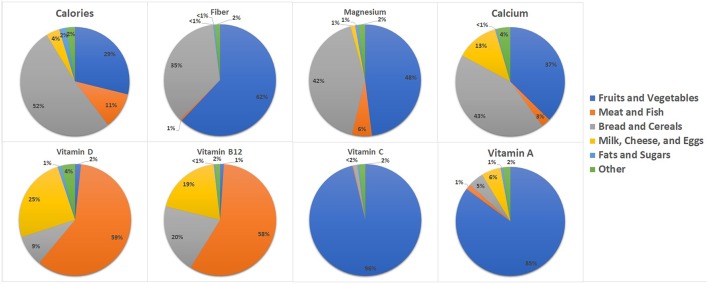
Breakdown of nutrient loss from avoidable food waste by food group.

When assessing the nutritional value of wasted food, it is important to look beyond calories to consider a range of nutrients required to support overall health. A focus on food quality rather than food quantity is critical. In this study, fruits and vegetables contributed 66% of total avoidable food waste. The nutrients derived from fruits and vegetables (e.g., fiber and Vitamin C) were higher relative to the other nutrients, as one would expect, but vitamin D (derived from meat and fish) was also important. For a full breakdown of nutrient loss by food group, please refer to Figure 6. In this sample, wasted fruits and vegetables contributed 62% of wasted fiber, 48% of wasted magnesium, 85% of wasted vitamin A and 96% of wasted vitamin C.

**Figure 7:**
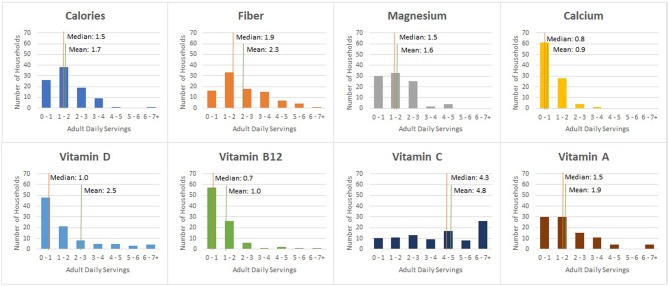
Household frequency of nutrient servings in weekly avoidable food waste.

The distributions of wasted nutrients expressed as daily adult serving requirements differ by nutrient and are presented in Figure 7. Based on this figure, we determined that the majority of households included in this study are wasting at least one daily adult serving of most nutrients analyzed.

## Environmental Analysis

### Global Warming Potential

**Figure 8:**
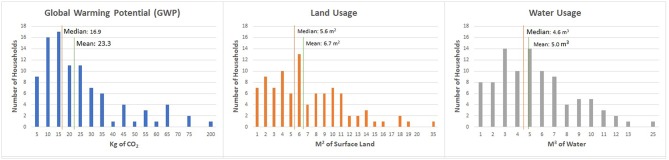
Household frequency of environmental impacts from weekly avoidable food waste.

The global warming potential (GWP) associated with avoidable household food waste calculated in this study was 23.3 kg CO_2_ per household per week, and the median value was 16.9 kg (Table 5). This equates to 1.2 tons of carbon dioxide from avoidable food waste per household per year, which is equivalent to one quarter of the emissions from a car being driven for a year, or 2.8 barrels of oil consumed based on a US Environmental Protection Agency calculator (45). The distribution of GWP (along with land and water use) per household per week is shown in Figure 8. Fruits and vegetables represented 66% of the total weight of the avoidable food waste and almost 40% of the CO_2_ generated by the total avoidable food waste. The meat and fish and milk, cheese, and egg categories represented a larger share of the CO_2_ generation than they represented of the total weight.

**Table 5:** Environmental impacts of avoidable food waste.

	**GWP** **(kg CO_**2**_)**	**Land** **(m^**2**^)**	**Water** **(m^**3**^)**	**GWP %**	**Land %**	**Water %**
Full basket	23.3	6.7	5.0	100.0%	100.0%	100.0%
Fruits and vegetables	9.2	2.0	1.9	39.3%	29.1%	38.0%
Meat and fish	7.8	1.8	1.2	33.6%	26.8%	24.6%
Bread and cereals	3.9	2.4	1.4	16.7%	35.6%	28.1%
Milk, cheese, and eggs	1.7	0.3	0.2	7.1%	4.8%	3.7%
Fats and sugars	0.4	0.1	0.1	1.7%	1.2%	1.5%
Other	0.4	0.2	0.2	1.6%	2.5%	4.1%

### Land Usage

Avoidable household food waste was estimated to be equivalent to 6.7 m^2^ of land per household per week. The median value was 5.6 m^2^. On an annual basis, the land used to produce avoidable food waste equated to 348.0 m^2^ per household per year. Once again, avoidable waste from fruits and vegetables represented the largest share of land use but was a lower proportion of land use relative to the proportion of total avoidable food waste weight. Avoidable waste from breads and cereals represented a larger share of land use than of weight. Meat and fish also represented a substantial proportion of the land used to produce avoidable food waste.

### Water Usage

Water usage associated with avoidable household food waste was calculated to be 5.0 m^3^ per household per week (the median value is 4.6 m^3^), or the equivalent of 260.0 m^3^ annually. This means that the avoidable food waste generated by a household represented 5,000 liters of water use weekly and 260,000 liters annually. The average 5-min shower uses 35 liters of water, which equates to 7,429 showers per year (46). Fruits and vegetables, meat and fish, and milk, cheese and eggs all contributed substantially to the water used to produce avoidable food waste.

## Discussion

### Overview

Overall, the sample of households that we audited in this study generated similar amounts of food waste to a more extensive sample in the same locale, discussed in Parizeau et al. (14), and similar food group proportions were observed in a randomly-selected sample from Guelph (unpublished data). The per capita total food waste generation rate in our sample of 1.1 kg per week, or 57.2 kg per year, is lower than the 85 kg per year estimate of per capita total residential food waste generated by the Commission for Environmental Cooperation (12). However, the CEC estimate is not based on actual audit measurement of household food waste. Despite the difference in values, per capita waste generation remains unacceptably high.

Our analysis has demonstrated that avoidable household food waste can be understood from multiple perspectives, and that diverse framings highlight different aspects of the food waste problem. A summary of the results from our analysis can be found in Table 6. We posit that these diverse framings can support the creation of evidence-based story-lines that foreground the issue of food waste in Canada. We now turn to a discussion of the potential discursive implications of our analysis.

**Table 6:** Summary of economic, nutritional and environmental impacts of avoidable household food waste.

	**Weight of edible food waste**	**Economic**	**Environmental**			**Nutritional**							
		**Cost**	**GWP**	**Land**	**Water**	**Calories**	**Fiber**	**Magnesium**	**Calcium**	**Vitamin D**	**Vitamin B12**	**Vitamin C**	**Vitamin A**
Fruits and vegetables	65.5%	52.0%	38.0%	29.1%	38.0%	28.9%	62.0%	48.0%	37.4%	1.7%	1.1%	96.3%	85.2%
Meat and fish	6.0%	12.7%	24.6%	26.8%	24.6%	10.8%	0.5%	5.8%	2.7%	59.3%	57.7%	0.2%	1.6%
Bread and cereals	24.2%	25.1%	28.1%	35.6%	28.1%	51.6%	35.4%	42.0%	42.9%	9.0%	20.0%	1.2%	4.6%
Milk, cheese, and eggs	2.1%	3.9%	3.7%	4.8%	3.7%	4.0%	0.1%	1.3%	12.6%	24.9%	19.3%	0.0%	5.8%
Fats and sugars	0.5%	0.7%	1.5%	1.2%	1.5%	2.1%	0.5%	0.9%	0.4%	1.1%	0.4%	0.4%	0.7%
Other	1.6%	5.6%	4.1%	2.5%	4.1%	2.6%	1.5%	2.0%	4.1%	4.0%	1.5%	1.9%	2.2%
Total	100.0%	100.0%	100.0%	100.0%	100.0%	100.0%	100.0%	100.0%	100.0%	100.0%	100.0%	100.0%	100.0%

A focus on the total weight of avoidable food waste in each household highlights that there is a relatively small group of households that waste very high amounts of food. It may be worth targeting this sub-group with interventions in order to address the hotspots of avoidable food waste in the residential sector. For example, messaging that focuses on the upper range (i.e., Q4 means or upper-end values) of health, economic, and environmental impacts may be more effective in convincing high-wasting households to change their waste-related behaviors, rather than exclusively focusing the overall mean impacts in these realms. In this case, the economic impacts could be framed as follows: “The average household wastes $936.52 buying food they do not eat each year, but some households lose over $1,600 annually through avoidable food waste.” The variability of food wasting habits across our sample also suggests that there are varying levels of food skills in these households that may be associated with food waste generation [e.g., meal planning, shopping, preparing and storing food: (14, 47–51)]. Targeted interventions focused on improving these food-based skills could help to change these behaviors among high-wasting households.

Looking at household food waste through the lens of a composition audit reveals that the proportions of wasted food across food groups are fairly consistent from low- to high-wasting households. Fruits and vegetables make up most of the avoidable food waste generated by all households, followed by bread and cereals, and then meat and fish. Other categories were much smaller in comparison. The prevalence of wasted fruits and vegetables is concerning from a nutritional perspective, as produce is a common source of key nutrients. This finding suggests that wasted food is a health policy issue: households are routinely purchasing large amounts of nutritious produce that they are not consuming. The very high rates of avoidable fruit and vegetable waste also have environmental and economic implications, as noted in Table 6. The prevalence of fruits and vegetables in the waste stream suggests that targeting these food groups may be a high policy priority to reduce total food waste volumes. We note that Cooper et al. (16) conducted a similar analysis using UK food waste data. Although the food waste categories differ between our two studies, Cooper et al. (16) found fruits and vegetables to be a smaller proportion of total edible food waste than was found in this study. The food categories fresh vegetables and salad, fresh fruit, and processed vegetables and salad contributed a total of 34% of total edible food waste (16), compared to our 66%. Differences in data collection methods, food preferences, shopping habits and definitions of edibility may account for these different results.

The significant relationships between the amount of avoidable food waste produced in a household and both total spending on food and number of items found in the waste stream indicates that over-purchasing is a major driver of avoidable food waste. People who bought high volumes of food or who bought a high diversity of items generated more wasted food. This may be the result of various household shopping and cooking practices, such as experimentation or impulse buying at the store. It might also suggest that more diversity in the diet creates a greater volume of waste. This finding speaks to the importance of influencing individual shopping behaviors in order to reduce food waste, but also in addressing the factors that encourage consumers to buy more in the first place, including retail strategies and broader cultural norms that support over-consumption.

We have framed our results as a list of most wasted items in order to make the sometimes nebulous issue of food waste seem more tangible. The list includes very common items in Canadian grocery baskets, enabling individuals to better imagine their own purchasing, consumption, and wasting behaviors around these specific items. The list also points to implications for retailers who make decisions about promotions, discounts, packaging, and in-store reprocessing of commonly wasted foods, which may impact consumer choices and behaviors (52–54). The list format allows for discursive framings centered on high-volume wasted items, while the economic, health and environmental analyses allow for framings focused on high-impact wasted foods.

### Economic Analysis

The economic analysis indicates that households spent an average of $936.52 per year on avoidable food waste. This is the equivalent of 16% of the average Canadian household expenditures on food bought from stores in 2017 [$5,934; (55)]. Meat and fish are relatively expensive foodstuffs, and so had a disproportionate impact on the cost of wasted food. As expected, households wasted a small overall amount of these high value proteins, but it is clear that interventions to reduce protein waste would have an important economic benefit for households.

Our analysis suggests that the study households wasted $18.01 per week on avoidable food waste. Another commonly cited value for food waste in Canada is $28 per week, but this estimate was not based on audits and likely included the value of unavoidable waste (56). In fact, $18.01 scaled to represent the total weight of both avoidable and unavoidable food waste would create a value of $27.29 per week. The National Zero Waste Council undertook efforts to quantify waste in Metro Vancouver to support their Love Food Hate Waste campaign. They estimated that an average household wastes more than $1,100 in edible food per year. This equates to $21.12 per week. The difference in value is likely attributable to food cost differences between Vancouver and Guelph.

### Nutritional Analysis

From a health perspective, our analysis shows that households have access to a breadth of nutrients that they are not consuming, including fiber, vitamins, and minerals. This is an especially concerning finding given the rates of inadequate intake in typical Canadian diets of fiber and many vitamins and minerals including vitamin B12, vitamin A, vitamin D, vitamin C, calcium, and magnesium. Furthermore, the prevalence of inadequate intake of calcium is a particular concern for older adults (37), which is noteworthy given Canada's aging population and the importance of both calcium and vitamin D in supporting healthy bone growth and maintenance (57). The high amounts of wasted fruits and vegetables represent the loss of calories, fiber, vitamins and minerals. Bread and cereal wastes also represent lost calories, fiber, and vitamins, and minerals. Meat, fish, dairy, and egg wastes represent lost calories, vitamins and minerals. The framing of food waste as a potential health risk may be a compelling one for individuals and policy-makers.

Cooper et al. (16) also considered nutrients for which adequate intake was a concern among the UK population. Similar to our results, Cooper et al. (16) found fresh vegetables and salad to be the largest contributor by food group to wasted fiber. Dairy and eggs and bakery were found to each contribute 27% of wasted calcium. Our findings reveal that 43% of lost calcium was from breads and cereals and 13% was from milk, cheese and eggs.

Some might argue that given the current global obesity epidemic (58), throwing out calories may not necessarily be a bad thing. However, focusing on calories alone does not accurately represent nutrient losses. According to Spiker et al. (59), focusing only on the caloric value of food waste risks over representing the influence of calorie-dense foods that typically carry fewer health benefits. This may result in nutrients of concern being overlooked. For example, nutrient-dense but low-calorie foods such as fruits and vegetables are better examined based on their nutritional quality rather than their caloric density.

Focusing on these nutrients presents a promising opportunity to develop nutrition-based narratives that would be relevant to most households. When food waste is contextualized as wasted servings of nutrients of concern, value and utility can be re-allocated to the wasted food. Had individuals consumed the foods in which these nutrients are commonly found, there would have been potential health benefits to be gained.

Similar to work conducted by Cooper et al. (16), our results support a mutual benefit to initiatives seeking to increase consumption (and therefore decrease waste) of fruits and vegetables. This is further supported by a study conducted by Black and Billette (60) suggesting that the majority of Canadians failed to meet Health Canada's 2007 recommendations for fruit and vegetable intake. Results presented in this study also support previous findings identifying wasted food as having a high influence on the availability of important micronutrients (59). If families can successfully lower their food waste generation by eating the fruits and vegetables they procure, they may also improve the quality of their diets.

It is worth noting that the nutritional numbers presented here are considerably lower than those reported in Conrad et al. (61). In that paper, authors estimated that the average American consumer wasted approximately 5,600 kcal per week, as compared to a weekly family waste generation of 3,366 kcal found in this study. However, Conrad et al. (61) did not measure food waste but estimated it based on aggregate USDA data. They found that fruits and vegetables were a much lower proportion of total waste than was the case in our data and that 422 grams of avoidable food waste was generated per day. This disconnect in results highlights the value of direct measurement of granular data to quantify both the volume and composition of household food waste. Conrad et al. (61) also suggest that healthier diets, as measured by the Healthy Eating Index, create more waste. This is based on estimates of the proportion of each food type wasted rather than on direct measurements of households with healthier diets. This hypothesis merits investigation at the household level as those who eat healthier diets may have a different propensity to waste. For example, Parizeau et al. (14) found that households with greater “food awareness” generated less organic waste.

### Environmental Analysis

Consumers tend to see food waste as an economic or social issue, more than an environmental issue (14, 47, 62–64). Given increased public attention to environmental issues of climate change, water, and agricultural land use in Canada, providing data to effectively communicate the environmental impacts of food waste may help to better frame this issue as a sustainability challenge in addition to a pocket-book issue. Our analysis reveals that avoidable household food waste is a substantial contributor to global heating, inefficient agricultural land use, and water loss. Cooper et al. (16) attribute 6.1 kg of CO_2_ from avoidable food waste per capita weekly in the United Kingdom. The average household in our sample had four occupants which means that our results from Canada (5.82 kg CO_2_ per capita per week) are consistent with those from the UK. The avoidable food waste water use estimates from our sample are lower than those from Cooper et al. (16), who suggest that avoidable food waste used 6.3 cubic meters per person per week. Our estimate is approximately one-quarter of that estimate.

### Methodological Strengths and Limitations

Methodologically, our study contributes a highly granular analysis of household food waste composition to the field of waste studies. We are unaware of any other published studies that have used this methodology with Canadian data, or that base their estimates of food waste impacts on direct observation of individual items in the residential waste stream. Our research also problematizes dietary research that relies on purchase records and self-reported consumption logs, but that does not assess the proportion of nutritious food that ends up in waste streams.

We note that there are limitations to this study design. A perennial methodological difficulty when studying household waste is the high variability both within and between households. The voluntary nature of participation in this study may have led to some self-selection bias. It is possible that only families with a pre-existing interest in and understanding of food chose to participate. Additionally, this study was time-intensive, requiring families to collect all grocery receipts, keep a 3-day food diary and withhold their waste for collection by the study team. These requirements may have served as a disincentive for families who were less organized, possibly correlating with poor food planning skills and higher food waste generation. Furthermore, participants were aware that their waste was being audited. Although this can result in some change in waste behaviors, it would have been difficult for families to hold back or attempt to hide their food waste in other waste streams as we collected all streams of waste in this study for an extended period of time. Furthermore, the City of Guelph is active in providing educational content on waste management topics through their website, the distribution of informational material to households, and participation in community events. As a result, community members may have a greater baseline understanding of food waste reduction strategies than people living in other communities. Another limitation is based in our relatively small sample size of 94 households. This is not representative of the socio-demographic diversity of the study city as a whole. Rather, this study focused on families with young children who had the time and interest in committing to this study.

## Conclusion

Our analysis is not only oriented to convincing householders and consumers that they need to change their perceptions and behaviors around food waste. Food waste is a systemic issue, and these framings are also meant to encourage systems-level interventions, including policy, regulations, infrastructure development, corporate practice, and culture change more broadly. Given the recent policy attention to food waste at different scales of government in Canada, it is clear that this issue will soon reach a stage of discursive institutionalization. However, there are still diverse and non-coherent discourses at play, and none have become hegemonic at this stage. We are not advocating for any one framing as the ideal discursive framing for food waste, but rather we encourage advocates, practitioners, and policy-makers to develop evidence-based communications and interventions. It is likely that multiple synergistic messages can support one another and allow for discursive framings that are diverse and tailorable for different audiences.

We acknowledge that this snapshot of the waste of families with children in Guelph is likely not generalizable to other contexts, although we encourage other researchers to conduct similarly specific and extensive observations so that we can collectively generate high quality information about the state of household food waste.

## Data Availability

The datasets for this manuscript are not publicly available because of restrictions related to our Research Ethics Board requirements. Requests to access the datasets should be directed to MM, mvonmass@uoguelph.ca

## Author Contributions

MM and KP contributed to the conception and design of the composition audit. JH, AD, and DM designed the Family Food Skills Study. AW, NC, and MW organized the database used for this study. MW conducted data analysis. MM, KP, and MG interpreted the analyses. MM and KP wrote the first draft of the manuscript. MG and JH wrote sections of the manuscript. All authors contributed to manuscript revision, read, and approved the submitted version.

### Conflict of Interest Statement

The authors declare that the research was conducted in the absence of any commercial or financial relationships that could be construed as a potential conflict of interest.

## Footnotes

^1^http://www.wrap.org.uk/content/household-food-waste-collections-guide.

^2^https://hannaone.com/category/food-infohistory/weight-equivalents.

^3^https://food-nutrition.canada.ca/cnf-fce/index-eng.jsp.
